# A multi-modal exploration of heterogeneous physico–chemical properties of DCIS breast microcalcifications†

**DOI:** 10.1039/d1an01548f

**Published:** 2022-03-21

**Authors:** Sarah Gosling, Doriana Calabrese, Jayakrupakar Nallala, Charlene Greenwood, Sarah Pinder, Lorraine King, Jeffrey Marks, Donna Pinto, Thomas Lynch, Iain D. Lyburn, E. Shelley Hwang, Grand Challenge PRECISION Consortium, Keith Rogers, Nicholas Stone

**Affiliations:** Cranfield Forensic Institute, Cranfield University Shrivenham UK k.d.rogers@cranfield.ac.uk; School of Physics and Astronomy, University of Exeter Exeter UK n.stone@exeter.ac.uk; School of Chemical and Physical Sciences, Keele University Keele UK; Division of Cancer Studies, King's College London, Guy's Hospital London UK; Department of Surgery, Duke University Medical Center Durham NC USA; www.DCIS411.com San Diego CA USA; Thirlestaine Breast Centre, Gloucestershire Hospitals NHS Foundation Trust Cheltenham Gloucestershire UK; Cobalt Medical Charity Cheltenham UK

## Abstract

Ductal carcinoma *in situ* (DCIS) is frequently associated with breast calcification. This study combines multiple analytical techniques to investigate the heterogeneity of these calcifications at the micrometre scale. X-ray diffraction, scanning electron microscopy and Raman and Fourier-transform infrared spectroscopy were used to determine the physicochemical and crystallographic properties of type II breast calcifications located in formalin fixed paraffin embedded DCIS breast tissue samples. Multiple calcium phosphate phases were identified across the calcifications, distributed in different patterns. Hydroxyapatite was the dominant mineral, with magnesium whitlockite found at the calcification edge. Amorphous calcium phosphate and octacalcium phosphate were also identified close to the calcification edge at the apparent mineral/matrix barrier. Crystallographic features of hydroxyapatite also varied across the calcifications, with higher crystallinity centrally, and highest carbonate substitution at the calcification edge. Protein was also differentially distributed across the calcification and the surrounding soft tissue, with collagen and β-pleated protein features present to differing extents. Combination of analytical techniques in this study was essential to understand the heterogeneity of breast calcifications and how this may link crystallographic and physicochemical properties of calcifications to the surrounding tissue microenvironment.

## Abstract 2

Combined crystallographic and spectroscopic methods were used to investigate the heterogeneity of breast calcifications found associated with ductal carcinoma *in situ*, revealing distinctive patterns in protein distribution and mineral composition.

## Graphical abstract

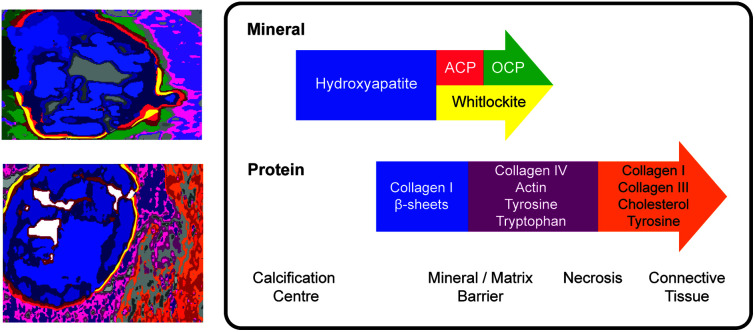

## 1. Introduction

Breast microcalcifications are a common feature of screening mammography and key diagnostic markers for breast disease. Mammographically, breast microcalcifications are classified based on distribution, size and morphology, with certain patterns more frequently associated with malignancy.^1^ Chemically, microcalcifications can be classified into type I and type II. Type I calcifications are mostly associated with benign breast conditions and consist of calcium oxalate dihydrate (COD, CaC_2_O_4_·2(H_2_O)). They are birefringent, translucent, and amber in colour. Conversely, type II calcifications are white, opaque, and non-birefringent, and consist primarily of hydroxyapatite (HAP, Ca_10_(PO_4_)_6_(OH)_2_), a calcium phosphate. Type II calcifications have been identified in both benign and malignant breast conditions.^2^

The introduction of mammographic screening led to a huge increase in ductal carcinoma *in situ* (DCIS) detection, with 85% of cases identified this way, whereas invasive cancers are still mostly identified by patients presenting with a palpable mass.^3^ DCIS has the potential to progress to invasive disease, which is thought to occur in 25–35% of cases.^4,5^ Currently, there are no robust markers to differentiate progressive and non-progressive cases of DCIS, leading to cases of overtreatment, therefore elucidating novel biomarkers for DCIS is a key step in improving patient outcome and experience.

Increasingly, studies have identified evidence for linking chemical and crystallographic differences in HAP calcifications to differing breast pathology groups. For example, carbonate substitution into the HAP lattice has been investigated using Fourier-transform *infra*-red (FTIR) and Raman spectroscopy, with higher carbonate levels associated with breast disease benignity.^6,7^ Additionally, studies using X-ray diffraction (XRD), found a higher HAP crystallinity in calcifications associated with invasive cancer compared to *in situ* and benign disease.^7–9^ Further, elemental analyses have revealed the presence of fluorine, sodium and magnesium in breast calcifications, with higher sodium levels being associated with malignancy.^10,11^

In addition, magnesium whitlockite (WH, Ca_*n*_Mg_3−*n*_(PO_4_)_6_(OH)_2_) has been noted as a secondary crystalline phase in breast calcifications. There are, however, conflicting reports on the association of whitlockite presence with malignancy, with some studies indicating whitlockite is a marker of invasiveness, while others report this phase as a sign of benignity.^7,8,11^ Furthermore, a handful of studies have suggested the presence of HAP precursor phases in pathological calcifications, such as amorphous calcium phosphate (ACP, Ca_9_(PO_4_)_6_) and octacalcium phosphate (OCP, Ca_8_(HPO_4_)_2_(PO_4_)_6_·5H_2_O).^12^ ACP was found associated with invasive calcification in one study and at the surface of DCIS calcifications in another.^13,14^ Contrarily, OCP has not been directly measured in breast calcifications previously but is suggested as a likely precursor in acidic conditions, and has been found in other pathological calcifications such as dental calculi.^15,16^ HAP and WH formation from these precursors has been suggested to be dependent on characteristics of the surrounding tissue microenvironment, including factors such as ion presence and pH.^9^ For example, magnesium ion presence has been shown to impede HAP formation, while an acidic pH and magnesium ion excess will enhance the formation of WH over HAP.^16,17^

Previous studies have shown associations between protein expression and calcification presence in breast tissue. For example, proteins linked to bone formation such as bone morphogenetic protein 2 (BMP-2) and alkaline phosphatase (ALP) have proposed involvement in calcification formation processes.^18,19^ Additionally, collagen is a protein of interest in pathological calcification due to its role in guiding bone mineralisation. Studies have found collagen was not interspersed with calcification, rather surrounding the calcification or the calcifying duct, depending on the tissue pathology.^7^ Contrarily, non-collagenous proteins (NCPs), were found interspersed with the mineral component in DCIS calcifications.^11^ Further, studies using FTIR demonstrated a changing amide: phosphate ratio in calcifications found in differing breast tissue pathologies, with higher levels of protein associated with disease invasiveness.^6^

Many of the aforementioned studies demonstrated the independent capabilities of XRD, Raman and FTIR spectroscopy as tools to determine key breast calcification features. Additionally, Kunitake *et al.* have shown the power of combining techniques such as Raman, second harmonic generation and histological staining in creating accurate pictures of calcification chemistry and soft tissue features.^11^ For the first time, this study combines diffraction and spectroscopic techniques to provide a unique commentary on the benefits and limitations of a multimodal approach. Further, this study highlights the heterogeneity of individual calcifications both within the calcification itself and between calcifications found in the same patient and same pathology. This will provide an essential basis for investigative studies into the calcification landscape.

## 2. Experimental

### 2.1. Samples

Two formalin-fixed paraffin embedded (FFPE) breast biopsy samples from separate patients from Duke University were selected for analysis. Ethical approval was received from NHS Health Research Authority, REC number 18/LO/0945 and the Duke University Health System Institutional Review Board (DUHS IRB), protocol number Pro00054515 and from the ethical committees at Cranfield University and the University of Exeter. A waiver for consent was obtained as part of the IRB approval, with all personal identifiers removed before undertaking this study.

Both samples were high-grade DCIS, with no concurrent invasion or known recurrence. Sample 1 had micropapillary features associated with the DCIS, while Sample 2 contained comedo and cribriform DCIS features. Both samples contained multiple calcifications associated with DCIS, and five were randomly selected for detailed mapping analyses, two from Sample 1 (1A and 1B) and three from Sample 2 (2A, 2B and 2C)

Four sequential sections were cut from each sample block, one of 2 μm thickness and three of 5 μm thickness; for FTIR spectroscopy, Raman spectroscopy, haematoxylin and eosin (H&E) histological staining and XRD respectively.

### 2.2. H&E

**Fig. 1:**
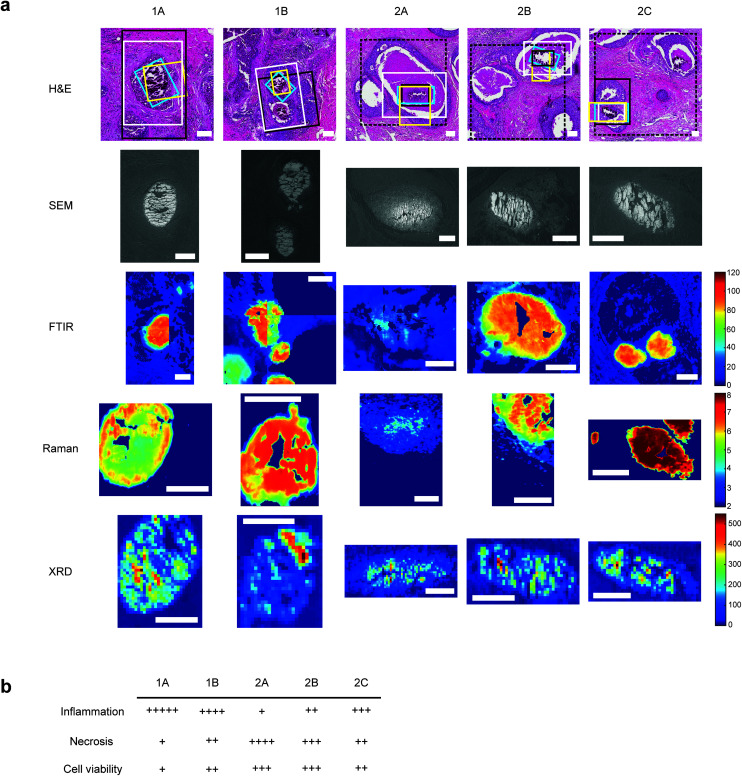
Multimodal mapping approaches for breast calcification investigation. (a) H&E stains of the calcifications investigated with regions of interest for each technique marked for SEM (white), FTIR (black), Raman (yellow) and XRD (blue). Where FTIR mineral and protein areas differ, FTIR protein ROIs are marked with a dashed black line. FTIR images show the peak intensity for the phosphate peak, Raman images show phosphate/amide ratio and XRD maps show total crystalline material present, calculated from total diffracting area. Scale bars = 150 μm. (b) table showing a summary of key histological features quantified relative to the five calcifications.

For each sample, a 5 μm section was mounted onto a standard glass slide and stained with H&E staining (Fig. 1a). Histopathological features local to each calcification were identified by expert pathologists at King's College Hospital NHS Foundation Trust, including extent of necrosis, associated inflammatory cells, surrounding cell viability and level of fibrosis.

Qualitative analysis of H&E-stained slides indicated evidence of inflammatory cells in the vicinity of all calcifications, with Sample 1 having higher levels of inflammation than Sample 2.

1A had limited epithelial cell viability, with a large volume of epithelial nuclear debris present and limited necrotic tissue. 2C had a higher level of viable epithelial cells, with some debris close to the limited necrosis within the duct. Similarly, 1B was mostly surrounded by viable epithelial cells, with necrosis levels similar to 2C. Contrarily, 2B had mostly viable epithelial cells, but high levels of intra-ductal necrotic debris. 2A showed similar characteristics, with the highest level of necrotic debris found in this duct. This is summarised in Fig. 1b.

### 2.3. FTIR

A 2 μm section from each sample was mounted on barium fluoride (BaF_2_) slides for FTIR analysis. Hyperspectral images were obtained directly from paraffinized tissues using an Agilent FTIR spectral imaging system (Agilent 620 FTIR microscope coupled with an Agilent 670 FTIR spectrometer, Australia).

Mid-IR light (2–12 μm) from a Globar® light source was transmitted through the sections using a 15× Cassegrain objective and collected using a matched condenser onto a liquid nitrogen cooled 128 × 128 pixel mercury cadmium telluride (MCT) focal plane array (FPA) detector. A single FPA tile on the detector gives an image of 704 × 704 μm^2^ and several tiles were measured in a mosaic to cover the regions of interest that included the calcification and the surrounding soft tissue for each of the 5 samples. Each image covered an area of around 2.2 × 2.5 mm^2^ at a pixel resolution of 5.5 × 5.5 μm^2^. IR spectra were collected in the range of 900–3800 cm^−1^ at a spectral resolution of 4 cm^−1^ averaged over 32 scans per pixel. For each image, a background signal was collected using a clean area of the BaF_2_ substrate which was then averaged out of the sample signal. The background was collected using the same imaging parameters however averaged over 128 scans per pixel.

Single point spectral measurements were carried out on the same calcifications using the same instrument and objectives, by transmitting IR light through an adjustable aperture onto calcifications and collecting light using a single point MCT detector. Aperture size was proportional to the calcification size. Point spectra were measured in the spectral range of 600–2000 cm^−1^ at a spectral resolution of 4 cm^−1^ averaged over 32 scans. Background measurements for each point was collected in the same spectral range at 4 cm^−1^ spectral resolution averaged over 128 scans.

### 2.4. FTIR Data Analysis

Raw IR spectra were pre-processed by truncating the spectral range to 900–1800 cm^−1^, then baseline corrected using a 4th order polynomial and min-max normalised. The pre-processed spectra from each image were independently subjected to *k*-means cluster analysis, which is a rapid way to segment the distinct spectral features. Different numbers of clusters (5–15) were tested for each image, to obtain the best possible segmentation of the histological classes. The segmented histological features were annotated by expert pathologists and compared to reference H&E images for further qualitative analysis of spectral features.

FTIR peaks were compared to spectra of standards of ACP, OCP, HAP and WH previously measured in our laboratory, in order to determine the mineral phases present.^20^ For protein analysis, spectra were compared to peak positions in published literature.^21,22^

### 2.5. Raman

For each sample, a 5 μm section was mounted on polished stainless-steel slides for Raman analysis.^23^ Due to interference of prominent paraffin peaks, samples were dewaxed using an optimised protocol previously reported using the chemical dewaxing agents Histoclear and isopropyl alcohol in decreasing alcohol proportions for 30 minutes, as follows.^24^ Three successive five-minute baths in HistoClear with gentle agitation were used to remove the paraffin, followed by a series of rehydration steps in graded Isopropyl alcohol.

Rehydration took place *via* ten sequential immersions in each of 100%, 90%, 70% graded isopropanol baths; followed by a final immersion in distilled water.

Raman spectroscopic experiments were performed with Renishaw InVia Raman system, in a temperature stabilised laboratory, minimising any drift during the measurements. An 830 nm laser source at 100% laser power, measuring 110 mW at the sample stage, 50× long working distance (NA 0.4) objective and a 600 l mm^−1^ grating were used. WiRE 4.1 software (Renishaw plc) enabled Streamline® mapping with a computer-controlled xyz stage and simultaneous charge shifting on the deep depletion CCD camera. Standard materials (silicon, fluorescent green glass and polyethylene) and a NeAr lamp calibration were applied to ensure Raman wavenumber shift was correct for each spectral acquisition, ensuring reproducible measurements. Spectra were collected and controlled by WiRE software, with a 15 s exposure time, ‘slalom setting’ on and centred at 1500 cm^−1^.

Initially, a microscopic scan coupled with a 5× objective was exploited to identify the same regions of interest (ROIs) detected using FTIR by comparing the orientation, position and presence of distinctive reference features. Raman maps were then attained with a 50× objective by raster scanning of the laser across the calcified section, by balancing the step-size acquisition and CCD y-bin to achieve image pixels of 1.4 × 1.4 μm^2^.

### 2.6. Raman data analysis

Data analyses were performed using the commercial software WiRE, MATLAB (MathWorks) and OriginPro2019 (Originlab Corporation). This included (i) pre-processing; (ii) unsupervised/statistical analysis, and (iii) curve fitting analysis.

Data pre-processing consisted of cosmic ray removal by applying a median filter using WiRE. The hyperspectral images were subsequently subjected to multivariate statistical investigations by principal component analysis (PCA) and *k*-means cluster analysis in MATLAB.

PCA is an unsupervised classification method, which reduces the dimensionality of the original image data by discriminating small variations between spectra, enclosed in the principal components (PC). Visualization of this spectral segmentation is provided by the loading plots and related score distribution maps. Performing PCA was an *a priori* approach to visualize a map in a space of small dimensions, without losing the richness of information of the original measurement.

Raman maps were decomposed by *k*-means clustering approach, which groups the spectra according to their similarity, forming *k*-clusters, each one representing the mean spectrum from different regions of the map with the same molecular properties. Distribution of the clusters is visualised in the original image constructed with a thematic (false colour) map, which contains a classification based only on spectral information.

To obtain a clearer representation of the underlying biochemical composition of each *k*-means cluster, independent centroids (which represents the mean spectrum of each cluster) were decomposed using curve fitting analysis in OriginPro2019. Raman spectra were truncated in the spectral range of interest for phosphate (905–988 cm^−1^), carbonate (1013–1100 cm^−1^), and organic bands. A baseline correction by rubber-band method was performed for each region of interest before the following decomposition of bands, using the ‘Multiple Peak-fit’ function in OriginPro.

The investigation was performed using the Voigt function, which is the convolution of a Gaussian function and a Lorentzian function; where *y*_0_ = offset, *x*_c_ = centre, *A* = area, WG = Gaussian full width at half maxima (FWHM), *W*_L_ = Lorentzian FWHM, *t* = wavenumber, *n* = number of peaks:
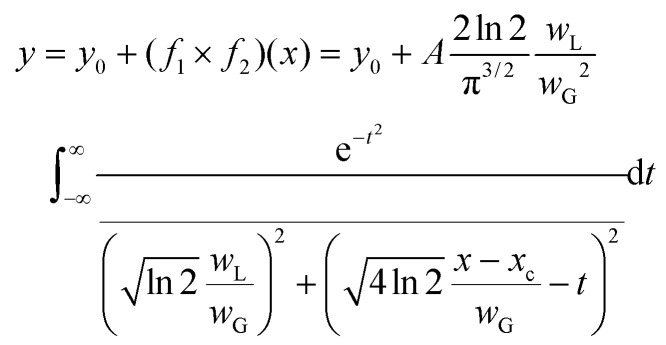


The peak central region and peak width parameters were fixed and released during fitting to help with initializing the parameters. The iteration procedure was stopped when the best fit was achieved (reduced chi-square <1 × 10^−9^).

Curve fit analysis was performed for the standard minerals of OCP, ACP and WH, previously measured with Renishaw InVia Raman, thus defining the discriminant peaks of PO4^3−^ and CO3^2−^ used to interpret the presence of these calcium phosphate precursor phases in this study. Information provided from peak decomposition on the sub-bands of the phosphate band (960 cm^−1^) and carbonate band (1070 cm^−1^), was used to discriminate carbonated HAP (CHAP) from ACP or OCP, which have a similar peak profile, and to evaluate the carbonate content of these microcalcifications.

In particular, ACP is a metastable form of cHAP that does not possess a well-defined structure, stability, or crystalline form. For these reasons, ACPs typically exhibit a broad range of Ca/P ratios (usually between 1.15 and 1.67) depending on pH and ions in solution.

Due to their similar Raman peak decomposition profile, and the poor literature regarding the Raman analysis of ACP, ACP and cHAP were distinguished based on their sub-band differences, specifically concerning the vibrational modes of the phosphate groups of:

 • v1 PO4^3−^ band at 950–955 cm^−1^, (the prominence, the broader band and the shift to higher wavenumber is associated to cHAP peak profile)

 • v3 PO4^3−^ bands at 1029 and 1040 cm^−1^ (they increase in intensity and area for ACP)

 • v3 PO4^3−^ band at 1076 cm^−1^ (it increases in intensity and area for cHAP by enveloping the carbonate band at 1070 cm^−1^)

To evaluate the shape, content and position of the peaks for the phosphate and carbonate bands of these five samples, eight HAP powder standards with different amounts of carbonate (previously measured with Renishaw InVia Raman system) were analysed and decomposed using the curve fitting analysis, parameters and peak-fit functions previously described in OriginPro.

The position of the most prominent and discriminating PO4^3−^ and CO3^2−^ bands (960 cm^−1^ and 1070 cm^−1^ respectively) were assessed based on tables published in the literature, and the results of curve fitting analysis were used to set up a calibration curve for C : P ratio and subsequently applied to calculate the carbonate content of these five microcalcifications (ESI – Fig. 1).

### 2.7. XRD

A 5 μm section from each sample was mounted on 12.5 μm thick polyolefin substrate stretched over 38 mm diameter aluminium rings and held in place with rubber O-rings. XRD experiments were carried out at the i18 beamline at Diamond Light Source, Didcot, UK, using a beam energy of 12 keV and spot size of 5 × 5 μm^2^. Samples were mounted at 90° to the X-ray beam and FTIR and H&E images were used to identify the ROIs. X-ray fluorescence (XRF) mapping at 4.5 keV with an Xpress3 detector, was used to confirm the exact position of calcifications relative to the X-ray beam. XRD maps were collected in continuous mode using a step size of 11 μm, measuring for 15 s per point, using an Excalibur detector.

### 2.8. XRD data analysis

1-D data was produced *via* azimuthal integration of 2-D detector images, and background subtracted using polynomial curve fitting using Diamond Analysis WorkBeNch (DAWN) 2.18.0 software (Diamond Light Source).^25^ Phase identification was carried out using the International Center for Diffraction Data (ICDD) database (PDF-4, 2018) and microstructural analysis was carried out using Topas 6.1 software (BRUXER AXS).

Coherence length, CL, is a valuable characteristic of crystalline materials, representing the average distance over which lattice order persists within a crystal, and often employed as a measure of ‘crystallinity’. CL can be quantified using the Scherrer equation:
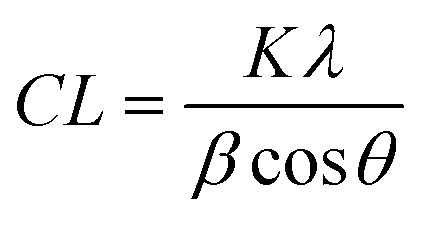
where *K* is the Scherrer constant (0.9), *λ* is the wavelength (0.1033 nm), *β* is the FWHM and *θ* is the Bragg angle.

The morphological anisotropy of biogenic hydroxyapatite crystals means this feature has been characterised in three directions, <00*ℓ*>, <*hk*0> and <0*k*0>, to assess the full extent of potential crystallinity differences. This was achieved using the 002, 004, 210 and 030 diffraction maxima.

Similarly, crystalline materials such as HAP consist of ‘unit cells’, the smallest repeating unit which contains the full crystal symmetry. Measuring the axial length of these unit cells, (termed ‘*a*’ and ‘*c*’), gives an insight into potential ion substitutions in the crystal lattice.

Parameterised data was plotted into maps using MATLAB R2020a (MathWorks).

### 2.9. SEM

Scanning electron microscopy (SEM) was carried out on the polyolefin and BaF_2_-mounted samples using a 51 mm aluminium stub, the stainless-steel mounted samples using a 127 mm aluminium stub. Images were collected using a Hitachi SU3500 system with an 11 keV beam energy and 70 Pa vacuum in variable pressure (VP-SEM) mode. A backscatter electron (BSE) detector was used for polyolefin and BaF_2_ slides and an ultra-variable pressure detected (UVD) for stainless steel slide to enhance contrast between the soft and mineralised tissue components and best account for charging effects due to the use of different substrates.

## 3. Results & discussion

### 3.1. Technique combination

For the first time, this study has combined Raman and FTIR spectroscopy with X-ray diffraction, histopathology, and SEM analysis to create a wide-scale, detailed picture of individual calcifications and their surrounding soft tissue in DCIS.

**Fig. 2:**
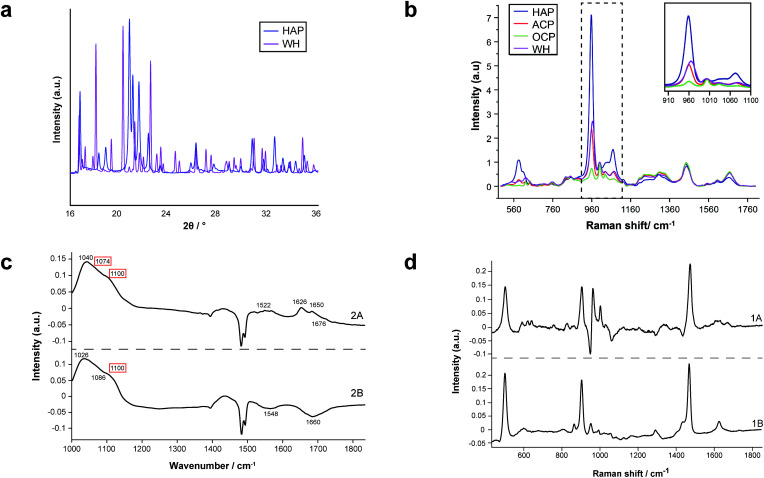
Standard patterns for calcium phosphate phases and PCA loadings. (a) Standard diffractograms of HAP and WH for XRD. (b) Standard spectra of HAP, ACP, OCP and WH for Raman. (c) PCA loadings for FTIR showing WH and OCP peaks (red boxes, WH and OCP cannot be separated using FTIR analysis, therefore are presented as a single image from PCA analysis). (d) PCA loadings for COD in Raman analysis.

Firstly, X-ray diffraction experiments provide characteristic diffraction patterns for crystalline phases such as HAP and WH, which can confidently be distinguished from one another (Fig. 2a). While this is also true of Raman and FTIR to some extent, the overlap of key peaks at around 960 cm^−1^ in the Raman data, which represents subtly different phosphate peaks from HAP, WH, OCP and ACP means extensive deconvolution is required to infer the phases present (Fig. 2b). In contrast, Raman and FTIR can identify transitional, low crystallinity phases such as OCP and ACP, which cannot be confidently identified using XRD in the range of angles interrogated in this work (Fig. 2b and c). Therefore, combination of the three techniques here is essential to fully understand the mineral make-up of breast calcifications.

Additionally, both Raman and FTIR spectroscopic analyses can identify carbonate presence in the HAP lattice structure due to the presence of key peaks and elucidate the extent of these substitutions by comparison with other peaks. Although this is not possible directly using XRD, it can be inferred using unit cell axial dimensions and coherence lengths as proxies.

Furthermore, Raman and FTIR can provide information on the protein composition (and other biomolecular composition) of the calcification and surrounding soft tissue, which is not found in the wide-angle XRD techniques employed in this study.

Therefore, the combination of Raman, FTIR and XRD is essential to fully understand the mineral and protein make-up of calcifications associated with DCIS and their surrounding soft tissues. In addition to this, the incorporation with histopathology allows the features of the minerals and proteins to be linked with histopathological features.

### 3.2. Mineral

**Fig. 3:**
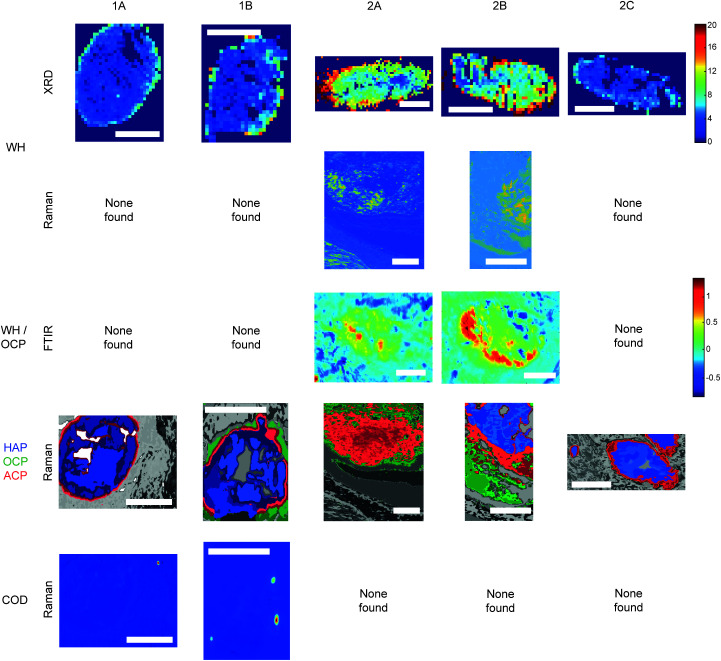
Additional phases maps across calcifications. Maps of calcifications showing distribution of whitlockite (WH), octacalcium phosphate (OCP), amorphous calcium phosphate (ACP) and calcium oxalate dihydrate (COD). Scale bars = 150 μm.

Raman, FTIR and XRD analyses indicated a higher relative amount of mineral towards the central region of the calcification compared to the edges (Fig. 1a). HAP, WH, OCP and ACP were all found in varying amounts and localised to different regions in the calcifications (Fig. 3).

#### 3.2.1. HAP is the primary calcification phase

**Fig. 4:**
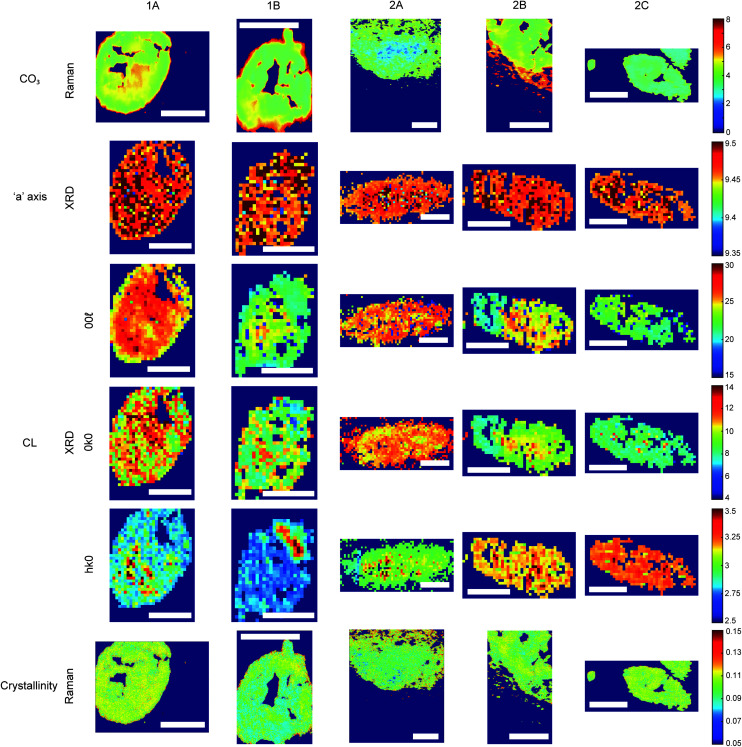
Hydroxyapatite feature patterns across calcifications. Maps of calcifications for total carbonate content (%) measured using Raman; length of the unit cell ‘*a*’ axis (nm) and coherence length (nm) measured using XRD; and crystallinity (cm) measured with Raman using 1/FWHM of the phosphate peak at 960 cm^−1^. Scale bars = 150 μm.

All analytical techniques showed the presence of HAP in all five calcifications (Fig. 3 and 4). This finding is not unexpected, as multiple studies have previously reported HAP calcifications in a range of breast pathologies, including DCIS.^8^

**Fig. 5:**
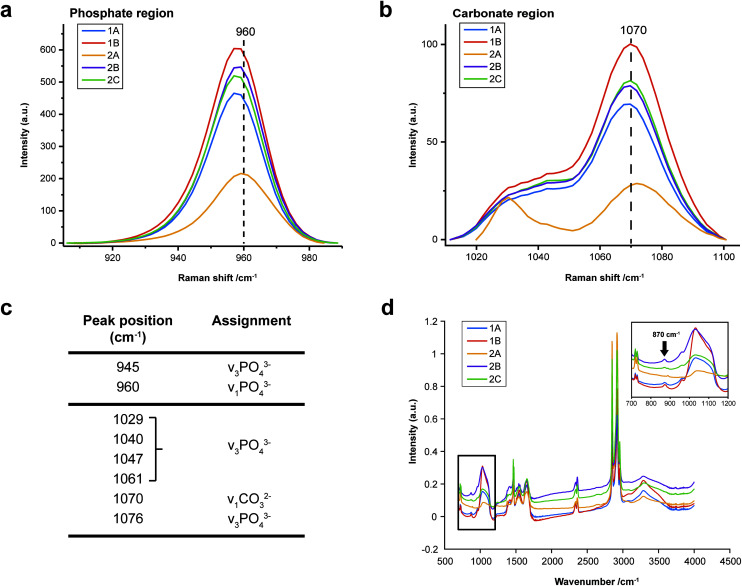
Carbonate identification. (a) and (b) Mean Raman spectra for each calcification around the phosphate peak at 960 cm^−1^ (a) and carbonate peak at 1070 cm^−1^ (b) used to identify mineral phases present and carbonate weight percentage. (c) Key peaks fitted in Raman analysis in the regions presented in (a) and (b). (d) Point spectra of each calcification from FTIR analysis. Single spectrum from each calcification showing the presence of B-type carbonate, evidenced by a peak at 870 cm^−1^. The spectrum from calcification 2A does not contain this peak.

FTIR and Raman spectroscopic analyses indicated that the HAP present contained B-type carbonate (BCHAP) substitution in all calcifications, except for 2A in FTIR (Fig. 4 and 5). Raman analysis found highest levels of carbonate substitution in 1B, decreasing 1B > 1A > 2C > 2B > 2A (ESI – Fig. 1†). For FTIR, the B-type carbonate substitution in the calcifications was deduced from the peak at 870 cm^−1^ using additional point measurements carried out in the 700–1800 cm^−1^ spectral range (Fig. 5d).^26–28^ For Raman, a peak at 1070 cm^−1^ was used to identify BCHAP presence (Fig. 5a–c). A-type carbonate (ACHAP) substitution was not found in any of these calcifications using either FTIR or Raman. In FTIR, ACHAP is usually identified through the presence of a peak at 878 cm^−1^. For Raman analysis, peaks at 1106 cm^−1^ and 1018 cm^−1^ representing carbonate and phosphate modes respectively are characteristic of ACHAP presence.^29,30^

Carbonate levels were found to be higher at the edge of the calcifications compared to the central region using Raman spectroscopy, with 1A, 1B and 2B showing similar levels and patterns of distribution. 2C only had a subtly increased carbonate level towards the calcification edge (Fig. 4).

XRD methods demonstrated that the ‘*a*’ axis of HAP unit cells was expanded compared to stoichiometric HAP (9.432 Å) in all calcifications. The ‘*a*’ axis was found to be further expanded towards the central region of calcifications compared to the edges (Fig. 4). 2A had an overall shorter ‘*a*’ axis compared to the other calcifications, which showed similar mean values for this parameter.

This ‘*a*’ axis expansion could be caused by numerous ion substitutions, such as carbonate, chloride, sodium or magnesium. Raman and FTIR identified B-type lower carbonate presence in the apatite towards the calcification centre, concurrent with a higher ‘*a*’ axis, therefore carbonate substitution may explain this pattern. However, there are often several concurrent substitutions in order to achieve charge balance, therefore carbonate is unlikely to be wholly responsible for the changing ‘*a*’ axis length.

Additionally, patterns of differing crystallinity were observed across individual calcifications. Raman spectroscopy, using 1/FWHM of the PO_4_ phosphate peak at 960 cm^−1^ to provide a relative measure of the mineral crystallinity, indicated a higher central crystallinity compared to the edges for 1A, 2B and 2C, but the opposite trend for 2A and 1B (Fig. 4).

XRD experiments were able to separate crystallinity (coherence length) based on crystallographic directions, thus coherence length was measured in three separate directions. CL parallel to the longest direction, <00*ℓ*>, was found to be higher towards the central region of calcifications for 1A and 1B. Samples 2A and 2C appeared to have relatively uniform CL measurements across the calcification, and 2B showed two distinct regions of CL, a low CL at the left-hand region and a high CL at the right-hand region of the calcification, with both regions showing little variation (Fig. 4).

Conversely, in the <0*k*0> direction, 1A, 1B and 2C showed no distinctive pattern, while 2A demonstrated a higher CL towards the edge of the calcification compared to the central region. The two regions of 2B observed for CL in <00*ℓ*> are also highlighted in the <0*k*0> direction, with the left-hand portion having a lower CL compared to the right-hand portion. The right-hand portion also showed a higher CL in the central region compared to the edge. This may suggest that the two areas of this calcification have formed at different times, merging into a single calcification at a later point.

Finally, in the <*hk*0> direction, CL was higher centrally compared to the calcification edges in 1A, 1B and 2A, while CL was uniform across calcifications 2B and 2C (Fig. 4). This higher central crystallinity is concomitant with lower carbonate substitution in this region and suggests the HAP in the calcification central region is more mature than that at the calcification edge.

#### 3.2.2. Additional calcium phosphate phases

XRD analysis also indicated the presence of WH in all calcifications, with highest amounts found in 2A, decreasing 2A > 2B > 1B > 1A > 2C. Raman analysis only indicated the presence of WH in 2A and 2B while FTIR analysis also suggested the presence of WH or OCP in 2A and 2B. All three techniques found WH signatures confined closer to the calcification edges with low or no WH in the central region of the calcifications (Fig. 2c and 3).

Raman analysis also deduced the presence of OCP in 1B, 2A and 2B at the apparent mineral/matrix barrier (Fig. 3). Further, FTIR indicated the presence of OCP or WH in 2A and 2B (Fig. 2c and 3). WH and OCP exhibit similar IR peaks, therefore it is not possible to discriminate between these two mineral phases for this technique. In addition, Raman analysis demonstrated the presence of ACP in all five calcifications. Generally, ACP was confined to the edge of the calcification, close to the bulk of the HAP. However, 2A showed features of ACP across the entire calcification (Fig. 3).

For calcifications where HAP, OCP and ACP are all present, there is a clear trend from outside the calcification to the calcification centre from OCP to ACP to HAP (Fig. 3). The lack of ACP and OCP within the central region of the calcification indicates the precursor phases have transformed fully into HAP, whereas this transition has not yet occurred at the calcification edge. Typically ACP transitions directly into HAP or through a further precursor phase of OCP in certain environmental conditions (ACP > OCP > HAP), therefore the presence of OCP furthest from the calcification is an interesting observation.^17^ This may suggest an environmental change causing the formation of OCP as a secondary precursor.

#### 3.2.3. Microenvironment and mineral phases

It is well documented that invasive cancer cells have an acidic extracellular microenvironment compared to normal cells, and studies have indicated that this acidification of the surrounding environment also occurs in DCIS.^31,32^ Additionally, previous studies have identified HAP as the most stable calcium phosphate mineral at a neutral pH, which is the usual pH of cells in the human body.^17^ At an acidic pH, HAP becomes more unstable, causing the preferential formation of WH. Therefore, WH formation at the calcification edge could be caused by an acidifying extracellular pH as the tissue environment changes through disease progression. In addition, OCP presence as a precursor phase is more likely at acidic pH, therefore increasing acidity may also have a role to play in OCP formation.

Both OCP and WH have been identified in 2A, 2B and 1B, using a combination of the three techniques, perhaps suggesting a link between these two minerals, or a common causation, such as acidic pH. However, XRD identified lower amounts of WH in 2C and 1A, where OCP was not present. This may suggest additional factors affecting WH and OCP formation beyond a changing pH or a differential timeline for these minerals forming in reaction to environmental changes.

The level of necrosis surrounding each calcification increased in the same relationship as the amount of WH found using XRD, with the lowest amounts in 2C and the highest in 2A. Similarly, OCP was found in the calcifications with higher levels of necrosis (1B, 2A & 2B). This further supports the hypothesis of microenvironment acidity consequence, as lower pH can increase the expression and activity of carbonic anhydrases (CA) such as CAIX, which in turn leads to an increase in necrosis.^33,34^

Similarly to microenvironmental pH changes, magnesium ion (Mg^2+^) concentration is of interest when discussing both calcium phosphate deposition and breast cancer progression. Firstly, WH is more likely to form in an abundance of Mg^2+^. Mg-Substituted HAP is incredibly unstable, therefore WH formation is preferred, where magnesium stabilises the structure.^16,17^ Additionally, Mg^2+^ can be incorporated into ACP and OCP, stabilising their structures and preventing the formation of HAP.^35^ Mg^2+^ ions have been shown to be of importance in breast cancer progression, with low levels in early-stage progression increasing carcinogenesis and angiogenesis, and increasing levels in later progression, enhancing metastasis of neoplastic cells.^36^ As calcifications form, the changing Mg^2+^ concentration will impact the calcium phosphate phase being formed, initiating a change to WH formation and stabilisation of ACP, consistent with the presence of this phase at the periphery of calcifications, and not in the central regions.

#### 3.2.4. Calcium oxalate dihydrate

There is some evidence of small areas containing calcium oxalate dihydrate (COD) within the HAP calcifications in Sample 1, but not in Sample 2 (Fig. 2d and 3). This was found through Raman analyses, but not with FTIR or XRD. COD presence is more commonly associated with type I breast calcifications; therefore, this finding was unexpected when focussing on type II calcifications.^2^

### 3.3. Protein

**Fig. 6:**
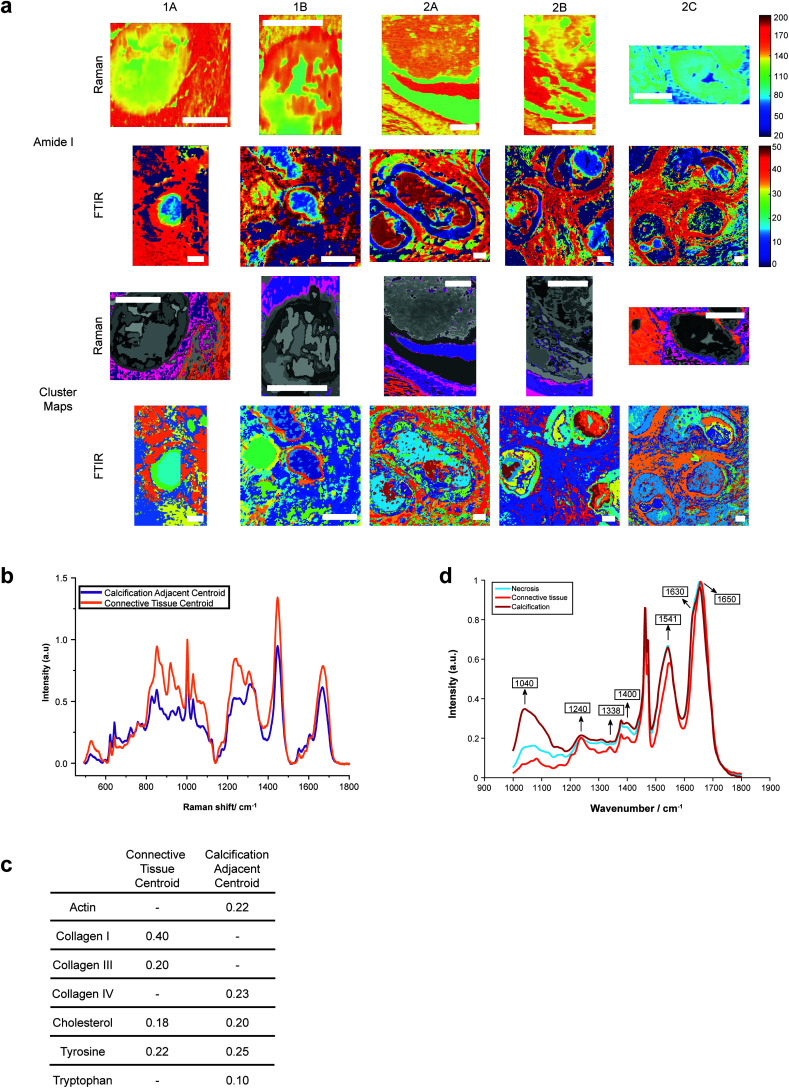
Protein distribution across calcifications. (a) Maps of amide I peak intensity and *k*-means clusters exhibiting protein signals from FTIR and Raman analysis. Scale bars = 150 μm. (b) Mean spectra for the calcification-adjacent centroid (pink/purple) and connective tissue centroid (orange) from Raman analysis. (c) Table showing relative contributions of summed spectra for each centroid in Raman analysis. (d) Mean spectra for the centroids associated with necrosis (blue), calcification (red) and connective tissue (orange) for calcification 2A from FTIR analysis.

Both Raman and FTIR spectroscopic analyses indicated the presence of proteins within and surrounding all five calcifications. The Amide I peak intensities for both techniques were highest in the soft tissue, and higher at the edges of the calcifications compared to the central region. Lower amounts of protein in the central region supports the claim of a mature mineral presence in this region. From Raman analysis, all calcifications appear to have similar Amide I peak intensities, except 2C which has an overall lower protein contribution (Fig. 6a).

#### 3.3.1. FTIR analysis

FTIR analysis was carried out on both the calcification and surrounding soft tissue and independent *k*-means clustering used on each image. The cluster centroid of the calcification region showed prominent peaks at 1040 cm^−1^, 1240 cm^−1^, 1338 cm^−1^, 1400 cm^−1^, 1541 cm^−1^, 1630 cm^−1^ and 1650 cm^−1^ (Fig. 6d).

The peak at 1040 cm^−1^ pertains to the phosphate vibrations directly associated with the calcification mineral in the gland, while the remaining peaks are associated with protein contributions. FTIR spectra were compared to the connective tissue centroid to investigate collagen I contribution. Peaks at 1240 cm^−1^, 1338 cm^−1^, 1541 cm^−1^ and 1650 cm^−1^ were identified as common features between the calcification centroid and the connective tissue centroid, indicating the presence of collagen type I within the calcification (Fig. 6a and d). This signature was also identified in the surrounding necrotic tissue.

In addition to the collagen features, FTIR peaks at 1630 cm^−1^ and 1670 cm^−1^ signified β-sheet protein conformations both within the calcification and in the surrounding necrotic tissue. Neither collagen type I nor β-sheet protein signals were identified in the surrounding epithelial regions.

#### 3.3.2. Raman analysis

Raman analysis carried out using independent *k*-means clustering on the calcification and surrounding soft tissue focused on three spectral regions: the amide I region (∼1666 cm^−1^); the amide III region (1229–1280 cm^−1^); and the C–C backbone region (850–920 cm^−1^). Centroids were compared to standard spectra of collagen I, II and IV, and key amino acids.

The centroid relating to connective tissue surrounding calcifications demonstrated a profile similar to that of collagen I and collagen III (Fig. 6a and b). There was also evidence of cholesterol and tyrosine signatures present in these centroids. A second centroid located close to the calcification within the necrotic tissue and containing epithelial glandular cells did not show evidence of collagen I and III, but had collagen IV signatures, as well as actin, cholesterol, tyrosine, and tryptophan (Fig. 6c). This region was also associated with OCP mineral signatures, with the highest protein contributions in this centroid correlated with this mineral. These patterns were true for all calcifications measured in the connective tissue regions.

This is an important observation, as OCP has been suggested to have roles in biological mineralisation by encouraging osteoblastic differentiation and interaction with collagen and osteocalcin.^37,38^ Further, OCP has also been implicated in the inflammatory response, with the presence of OCP enhancing IL6 production and macrophage migration.^39^

The Amide I region was further investigated using the second derivative of the peak at around 1666 cm^−1^, in order to determine protein conformational changes indicated by shifts in this peak position. The centroid representing the connective tissue had the highest wavenumber for the amide I peak at 1672 cm^−1^, suggesting a β-pleated sheet secondary protein structure, with the second centroid having a peak at a lower wavenumber of 1668 cm^−1^. Within the calcification itself, in the HAP portion, this peak was positioned at 1666 cm^−1^, signifying an α-helical protein structure.

For the majority of the calcifications measured, there was some level of protein association with the mineral portion, with highest amounts of protein towards the edge of the calcification. In the case of 2A, protein is associated with mineral across the entire calcification. Contrarily, in Raman analysis of 2C, there appears to be significantly less protein associated with the mineral portion of the calcification (evidenced by the small intensity of the protein Raman bands).

#### 3.3.3. pH and protein

Presence of proteins with β-pleated sheet features may potentially arise from misfolded proteins, occurring as a result of inflammation and oxidative stress. One such protein could potentially be the inflammatory protein, tumour necrosis factor alpha (TNF-α), though this assertion remains to be validated. TNF-α expression and activity could be impacted by the environmental pH. Studies have previously demonstrated a long-term reduction in TNF-α activity and expression with a more acidic pH in biologically relevant ranges.^40,41^ A reduced expression of TNF-α at lower pH is concurrent with the observation of weaker β-pleated signatures in the necrotic tissue compared to the mineral component.

Additionally, Raman analysis indicated the presence of the highest levels of β-pleated sheet proteins in the connective tissue centroid, decreasing towards the central region of the calcification, as indicated by the shifting of the amide I peak to a higher wavenumber. This peak shift could be associated with the changing conformation of collagen due to the action of matrix metalloproteinases (MMPs) on the extracellular matrix, consistent with previous observations from small angle scattering experiments.^42^ MMP collagen reorganisation plays an essential role in cancer cell migration and metastasis, therefore this find is particularly interesting as it may suggest early extracellular matrix changes in preparation for cancer metastasis, even at the DCIS stage. Studies have shown upregulation of certain MMPs in association with breast cancer, and these proteinases are also impacted by the surrounding extracellular environment, including pH. For example, MMP3 is upregulated in breast cancer, with increased expression at acidic pH, and has roles in cell apoptosis and epithelial–mesenchymal transition through collagen reorganisation in the breast.^43^

### 3.4. Slice by slice comparison

Ideally, this work would be carried out on the same section of tissue. However, the requirement for unstained sections and specialised substrates for each technique makes this impractical. Using serial sections was considered a suitable alternative, but we have demonstrated here that this may not allow pixel by pixel comparison between techniques.

Firstly, all five calcifications showed sectioning damage evidenced by the parallel ridges across the calcification surface and ‘dragging’ of mineralised material seen in SEM images (Fig. 1a). Additionally, some areas of calcification appear missing due to mineral loss in sample processing. This is particularly apparent in Sample 1 on the Raman stainless steel slides (see ESI – Fig. 2†).

Further, SEM images also highlighted the differences in calcification shape and size between the serial slices used for each analytical technique. These differences are most pronounced in calcification 1B, with Raman and XRD slides being most similar in this case, with the FTIR slide showing a somewhat different morphology. This is less evident in other calcifications, such as 1A (ESI – Fig. 2†).

Despite this, the work presented here has focused on overall patterns and characteristics across individual calcifications and between calcifications in tissue of a similar global pathology.

## 4. Conclusions

For the first time, we have combined spectroscopy and X-ray diffraction to elucidate the heterogeneity of DCIS calcifications. Key patterns have emerged which are consistent between the individual calcifications, but also important idiosyncrasies, reflecting both DCIS variability and its potential biomarkers.

One might hope, when commencing such a study, that each analytical technique might serve to complement or corroborate evidence from the other techniques. To a large extent this has been demonstrated and has enabled a detailed physicochemical snapshot of DCIS calcifications to be produced. This has enabled, albeit speculatively, new relationship models between calcification characteristics, formation mechanisms and the surrounding microenvironment.

## Author contributions

Sarah Gosling, Doriana Calabrese and Jayakrupakar Nallala contributed equally to this work. Sarah Gosling, Doriana Calabrese & Jayakrupakar Nallala: Writing – original draft, methodology, formal analysis, investigation. Charlene Greenwood: Writing – review & editing, supervision. Sarah Pinder, Lorraine King & Jeffrey Marks: Resources. Donna Pinto & Thomas Lynch: Writing – review & editing. Iain D Lyburn: Writing – review & editing. Shelley Hwang: Writing – review & editing, funding acquisition. Grand Challenge PRECISION Consortium: Funding acquisition. Keith Rogers & Nicholas Stone: Writing – review & editing, supervision, methodology, formal analysis.

## Conflicts of interest

There are no conflicts to declare.

## Supplementary Material

AN-147-D1AN01548F-s001

AN-147-D1AN01548F-s002
