# Supplementary material for: A multi-modal exploration of heterogeneous physico–chemical properties of DCIS breast microcalcifications
Source: Analyst. 2022 Mar 21;147(8):1641–54. doi: 10.1039/d1an01548f (PMC8997374; doi:10.1039/d1an01548f)
Supplement: AN-147-D1AN01548F-s001 [file AN-147-D1AN01548F-s001.pdf]

Electronic Supplementary Material (ESI) for Analyst.
This journal is © The Royal Society of Chemistry 2022

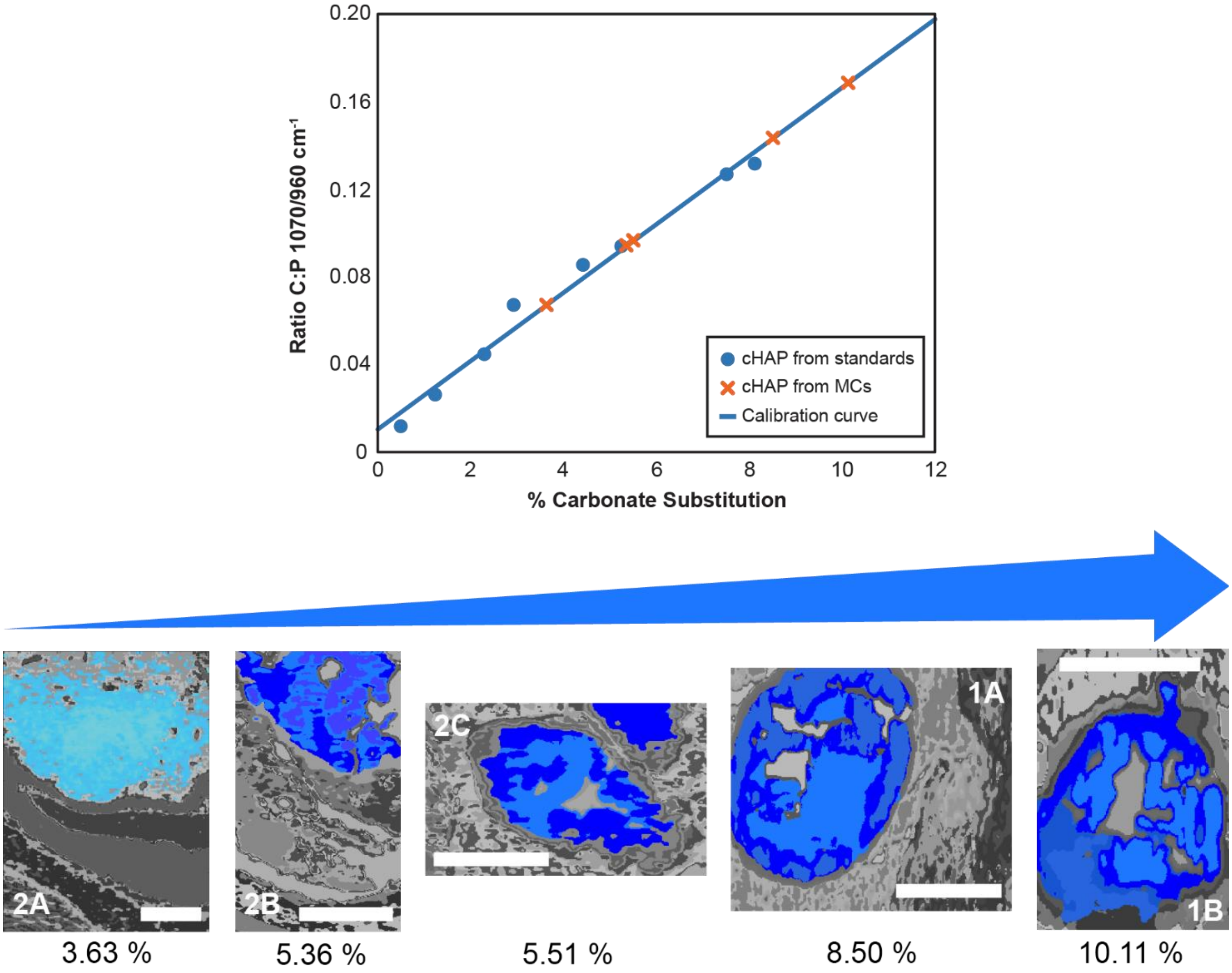

Supp. Fig. 1 Calibration curve for carbonate in Raman analysis. (top) Calibration curve constructed from measurement of HAP standards with known carbonate substitution, where the ratio of the areas of the 1070 cm^{-1} and 960 cm^{-1} peaks was measured and used to estimate carbonate substitution in each calcification. (bottom) location of carbonate HAP in each calcification with mean carbonate weight. Scale bars = 150 µm.
