# Supplementary material for: A multi-modal exploration of heterogeneous physico–chemical properties of DCIS breast microcalcifications
Source: Analyst. 2022 Mar 21;147(8):1641–54. doi: 10.1039/d1an01548f (PMC8997374; doi:10.1039/d1an01548f)
Supplement: AN-147-D1AN01548F-s002 [file AN-147-D1AN01548F-s002.pdf]

Electronic Supplementary Material (ESI) for Analyst.
This journal is © The Royal Society of Chemistry 2022

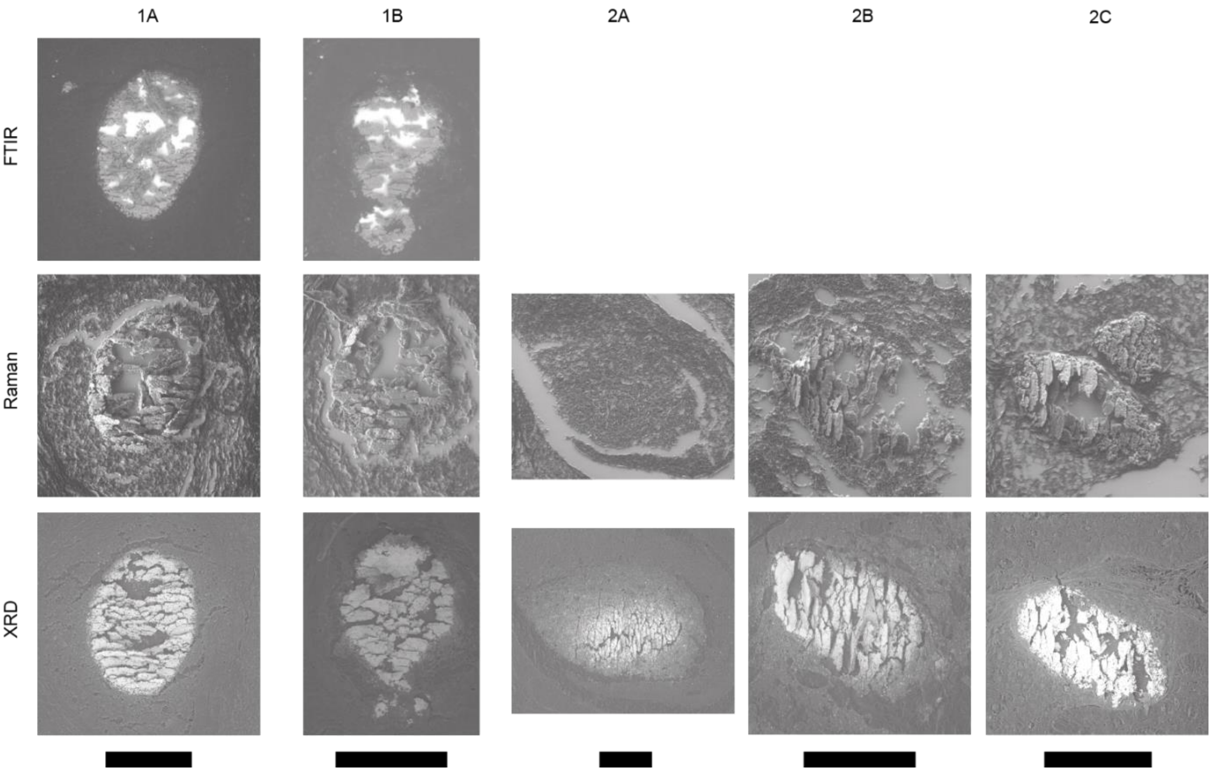

Supp. Fig. 2 SEM images of calcifications for all three analytical techniques. SEM images collected using a BSE detector for FTIR and XRD samples and a UVD detector for Raman samples to achieve the best level of contrast between the calcification, the surrounding soft tissue and the different substrates used for each technique. Images could not be collected for Sample 2 for FTIR due to slide damage. Scale bar (each column) = 200 µm.
